# Mobility of Vulnerable Elders (MOVE): study protocol to evaluate the implementation and outcomes of a mobility intervention in long-term care facilities

**DOI:** 10.1186/1471-2318-11-84

**Published:** 2011-12-16

**Authors:** Susan E Slaughter, Carole A Estabrooks, C Allyson Jones, Adrian S Wagg

**Affiliations:** 1 Faculty of Nursing, University of Alberta, Edmonton, Canada; 2 Faculty of Rehabilitation Medicine, University of Alberta, Edmonton, Canada; 3 Faculty of Medicine and Dentistry, University of Alberta, Edmonton, Canada

## Abstract

**Background:**

Almost 90% of residents living in long-term care facilities have limited mobility which is associated with a loss of ability in activities of daily living, falls, increased risk of serious medical problems such as pressure ulcers, incontinence and a significant decline in health-related quality of life. For health workers caring for residents it may also increase the risk of injury. The effectiveness of rehabilitation to facilitate mobility has been studied with dedicated research assistants or extensively trained staff caregivers; however, few investigators have examined the effectiveness of techniques to encourage mobility by *usual caregivers *in long-term care facilities.

**Methods/Design:**

This longitudinal, quasi-experimental study is designed to demonstrate the effect of the sit-to-stand activity carried out by residents in the context of daily care with health care aides. In three intervention facilities health care aides will prompt residents to repeat the sit-to-stand action on two separate occasions during each day and each evening shift as part of daily care routines. In three control facilities residents will receive usual care. Intervention and control facilities are matched on the ownership model (public, private for-profit, voluntary not-for-profit) and facility size. The dose of the mobility intervention is assessed through the use of daily documentation flowsheets in the health record. Resident outcome measures include: 1) the 30-second sit-to-stand test; 2) the *Functional Independence Measure*; 3) the *Health Utilities Index Mark 2 and 3; *and, 4) the *Quality of Life - Alzheimer's Disease*.

**Discussion:**

There are several compelling reasons for this study: the widespread prevalence of limited mobility in this population; the rapid decline in mobility after admission to a long-term care facility; the importance of mobility to quality of life; the increased time (and therefore cost) required to care for residents with limited mobility; and, the increased risk of injury for health workers caring for residents who are unable to stand. The importance of these issues is magnified when considering the increasing number of people living in long-term care facilities and an aging population.

**Trial Registration:**

This clinical trial is registered with ClinicalTrials.gov (trial registration number: NCT01474616).

## Background

Immobility is a major factor contributing to a reduced quality of life and preventable adverse events among older adults living in residential long-term care (LTC). Of the more than 150,000 older Canadians living in LTC facilities [1] almost 90% have some type of reduced mobility, [2] with approximately 40% of LTC residents with dementia losing their ability to walk annually [3,4]. Immobility leads to a loss of ability in activities of daily living (e.g. dressing or toileting), increased risk of falls and medical problems such as pressure ulcers and incontinence [5-9]. Although the adverse consequences of immobility and bed rest have been known for many years, elderly residents living in LTC facilities still commonly sit in wheelchairs or lie in bed for prolonged periods of time, in many cases for most of their waking hours [10,11].

When a resident loses the ability to stand up from a chair, the bed, or a toilet, both the time and the cost of health care escalate dramatically [12,13]. Transferring the resident then involves seeking a second health care aide to assist and often retrieving a mechanical lift to assist with the transfer. In the resource constrained environment of LTC, the extra time required for a transfer may translate to fewer transfers, significant delays in toileting (if toileted at all), and higher risk for a host of adverse consequences associated with immobility. Inactivity significantly affects quality of life for LTC residents in terms of ability to walk, balance and transfer [14-16].

Residents' immobility has ramifications on the nature of the work in LTC. Increased resident dependency increases the workload for health care aides as they perform more lifting and thereby heightens their risk of injury [17,18]. Approximately 60% of the loss of ability to walk in residents with dementia is potentially treatable [4]. The effectiveness of rehabilitation to facilitate mobility has been studied in LTC settings with dedicated research assistants or extensively trained staff caregivers [19-30]; however, the evidence that such rehabilitative approaches will be transferred, accepted, or sustained in the typically resource constrained environments of contemporary residential LTC facilities is lacking [31-33]. Few investigators have examined the effectiveness of techniques to encourage mobility by the usual caregivers in these settings, that is, by the unregulated health care aide workforce.

The primary purpose of this study is to determine if a simple mobility innovation (the sit-to-stand activity), implemented within the context of routine care activities of health care aides, can maintain or improve the mobility, function, and health-related quality of life of residents with dementia in LTC facilities.

We have selected our mobility intervention to be the sit-to-stand activity based on: a) the feasibility of integrating it into the daily practices of health care aides and residents using existing resources; b) preliminary evidence which supports integrating activity into functional situations such as dressing, toileting, or walking rather than introducing an exercise [27,34]; and, c) evidence suggesting that performance of the sit-to-stand activity may delay the well known trajectory of functional decline in LTC residents [24,32,34-38]. The sit-to-stand activity is thought to be one of the most mechanically demanding for LTC residents [39], yet it is still considered to be a low intensity exercise. Low intensity exercise has been found to improve physical performance [40,41] and activities of daily living [40,42,43] among frail older adults in LTC facilities although high intensity exercise did achieve a greater effect [40].

The benefits of implementing this simple sit-to-stand activity are seen at several levels. The intervention is low cost, does not require any specialized staff, training or equipment nor does it involve an important increase in the time required to care for residents; thus the mobility intervention possesses most of the attributes of successfully adopted innovations [44]. At the level of the health care aide and resident, standing up from a chair has the following attributes: relative advantage (does not require extensive training); compatibility (is consistent with activity in existing routines); low complexity (is a simple activity); trialability (can be easily tried and customized to individual residents); and, observability (improved mobility was visible to some health care aides during the pilot study).

Prior to the development of this protocol we completed a pilot study to assess the effect of the uptake of the sit-to-stand mobility intervention by health care aides on the mobility of LTC residents with dementia. This enabled us to: (1) assess the feasibility of integrating a simple activity into the daily work of health care aides; (2) develop a method of monitoring the adherence of health care aides to the activity; (3) calculate an appropriately powered sample size; and (4) begin to assess the response of this frail LTC population to the mobility intervention [34]. In the pilot study we assessed the mobility of 32 residents at baseline and after four months of exposure to the sit-to-stand intervention with the assistance of health care aides on day and evening shifts. Residents completed a mean of 29.7 (SD = 20.79; range 2 to 76) occasions of sit-to-stand repetitions in the month prior to the measurement of their sit-to-stand performance. For every 12 occasions of repeating the sit-to-stand exercise that month, the odds of improving or maintaining performance with the 30-second sit-to-stand test doubled (p = 0.02). This early finding with a small sample provides promising evidence that exposure to the sit-to-stand functional activity carried out in the context of daily care with health care aides may delay the loss of ability to transfer in nursing home residents with dementia [45]. We hypothesize that this ability to transfer will positively influence the resident's overall mobility, function and health-related quality of life.

Our research question is: What is the effect of the sit-to-stand mobility intervention on the mobility, function, and health-related quality of life of LTC residents with dementia?

## Methods/Design

**Figure 1:**
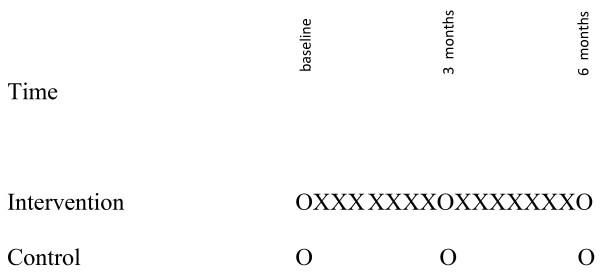
**Study Design**.

This is a longitudinal quasi-experimental study designed to demonstrate the effect of the sit-to-stand intervention on the mobility, function, and health-related quality of life of LTC residents with dementia (Figure 1).

### Sampling and recruitment

Residents with dementia residing in six LTC facilities in Edmonton, and the health care aides working with them, will be recruited to participate in the study. The purposive sample of research participants will be recruited from units which serve cognitively and physically frail older adults rather than units for young disabled people or sub-acute units. Residents that have a diagnosis of dementia and are able to transfer independently, or with the assistance of one person, will be eligible for inclusion in the study. Residents with a serious physical illness or life expectancy less than six months at the time of recruitment will be excluded from the study. We expect that most of the resident participants will be recruited to the study within eight months, however recruitment will continue throughout the study if necessary.

### Sample Size

The sample size calculation for the number of resident participants is based on the primary outcome measure in the pilot study: the number of sit-to-stands that the resident is able to complete in 30 seconds. Based on our earlier pilot work, we observed residents' ability to maintain and in some instances increase the number of sit-to-stands that they could complete in 30 seconds. We judge an increase of two sit-to-stands completed in 30 seconds to be a meaningful change in the residents' mobility. Based on existing research [10,38,46,47] and the pilot study, the standard deviation of the 30-second sit-to-stand measure is 3.5 sit-to-stands. Thus we will need 49 residents in each of the intervention and the control groups to detect an increase in the number of sit-to-stands that the resident is able to complete in 30 seconds (power = 0.80; 2-tailed; α = 0.05). Assuming an attrition rate of 20% over 6 months we will recruit 60 residents to the intervention group and 60 residents to the control group. We expect to succeed in recruiting and following our sample of 120 residents based on our experience with the pilot study and the experience of SES who recruited similar samples in two previous studies using similar recruitment methods [4,48].

### Intervention

Health care aides will be trained to prompt residents to repeatedly stand up and sit down on four occasions during daily functional activities (twice on each of the day and evening shifts). The number of repetitions on each occasion will vary according to residents' ability and fatigue. The sit-to-stand activity is to be integrated into usual care routines such as when entering the dining room at mealtimes, while toileting, and on other occasions of regular activity. The timing and location will be at the discretion of the health care aide. Health care aides will be reminded that, as with their usual care activities, when a resident's condition deteriorates they should consult with the registered nurse or licensed practical nurse about the appropriateness of repeating the sit-to-stand activity.

### Intervention group

Residents in the three intervention facilities will be exposed daily to the intervention for 6 months. This will enable an assessment of the effect of the intervention on residents' mobility over time and a preliminary assessment of the sustainability of the intervention. Their mobility, function in basic activities of daily living, and health-related quality of life will be measured at baseline, 3 months and 6 months.

### Control group

Resident participants in the control facilities will receive usual care. The only change in their routine will be the measurement of mobility, function and health-related quality of life on three occasions as for residents in the intervention group. Health care aides may work in more than one unit in a facility thus intervention and control groups will be designated at the facility level rather than the unit level to avoid contamination of the control group. It is also possible that some health care aides may work in more than one facility. We will assess the extent of contamination of the control group at the end of the study by asking health care aides who work control facilities if they have ever encouraged residents in the control facilities to repeat sit-to-stands.

LTC facilities in the intervention group will be matched to the control group facilities on ownership model (public, private for-profit, voluntary not-for-profit) and size because quality of care and resident outcomes are associated with ownership models [49-54] and size [48,49,52]. To assess the comparability of the intervention and control facilities a research assistant will gather information about each facility by: a) interviewing managers to obtain information such as staff-to-resident ratios on each shift, availability of rehabilitation services and size of units; b) observing activity across both the day and evening shifts using a structured observation schedule; and c) measuring environments using two standardized instruments: the Alberta Context Tool and the Professional Environmental Assessment Protocol (PEAP). Drawing on data collected from the observations and data elicited during the manager interviews, total PEAP scores will be calculated immediately following each manager interview.

### Schedule of Intervention

After obtaining informed consent the research assistant will gather residents' demographic information and measure residents' baseline mobility, function, and health-related quality of life in both the intervention and control groups (see Figure 1). The extent of baseline measurement is limited because of the frailty of this population and the likelihood of attrition during the study period. After baseline measurement the mobility intervention will be introduced exclusively to residents in the intervention group. Resident outcomes will be measured for both the intervention and comparison groups at three months and six months.

### Procedure

As the first residents are recruited from each intervention facility we will introduce the sit-to-stand intervention to the intervention facilities on the units where the residents reside. A nurse educator contracted for the study will deliver 20 minute education sessions on the sit-to-stand functional activity to as many health care aides as possible who are working on both day and evening shifts. These 20 minute sessions will be offered between 8 and 32 times depending on the size of the intervention facility. During these small group education sessions we will: a) describe the potential rationale for the sit-to-stand activity; b) provide standardized instruction and practice of the activity; and, c) illustrate how to document resident participation with the sit-to-stand activity. Following the education sessions health care aides will begin to facilitate the sit-to-stand activity with residents in their care who have been recruited to the study. Our pilot study demonstrated that health care aides require encouragement at regular intervals to consistently integrate the sit-to-stand activity into their daily routine. Thus we have included knowledge translation interventions that were tested in the pilot study to optimize the likelihood that residents in the treatment group will perform the sit-to-stand activity. In the first month the nurse educator will return to the intervention facilities to converse with the health care aides about their experiences with the sit-to-stand activity and to facilitate problem-solving around any barriers that they may have encountered. In the second month a set of reminders will be introduced including: a) prominently posting the names of resident participants where the change of shift occurs; b) affixing a colourful sticker to residents' bedroom doors and beside their beds; and, c) marking with a sticker the usual documentation flowsheets where the health care aides record residents' shift-to-shift responses to the exercise. During the third month 20 minute problem solving sessions with health care aides will be conducted to identify barriers to completing the sit-to-stand activity and to suggest ways to overcome them. The next month a poster and small handouts that summarize the discussion and problem-solving sessions will be posted on the treatment units and distributed to the health care aides. Each month a different approach will be taken to heighten the awareness of the health care aides about the importance of continuing to integrate the sit-to-stand activity into the residents' activities of daily living.

Health care aides will be invited to participate in an in-person, 30 minute interview pertaining to a specific resident if they have been permanent staff members in the facility and have been assigned to work directly with the resident in the past week. Although we considered blinding the research assistant to the intervention this is not possible to maintain because residents and health care aides will undoubtedly refer to the sit-to-stand intervention at some point during their contact with the research assistant. To minimize possible bias the research assistant gathering the outcome data will be independent of the nurse educator who will introduce the mobility intervention to the intervention group. The research assistant will not have access to data that were collected during the previous round of assessments and are unlikely to recall residents' precedent scores. The research assistant will assess all residents for the duration of the study unless unavailable; when the research manager will perform this duty. Therefore inter-rater reliability between the research assistant and the research manager will be assessed at the beginning, middle, and end of data collection.

The extent to which resident participants in the intervention group are exposed to the sit-to-stand activity will be measured using daily *documentation flowsheets *which are part the residents' health record. Managers expect these flowsheets to be completed by health care aides at the end of every day and evening shift. The precise method of incorporating the documentation of the sit-to-stand activity will be tailored to the existing flowsheets in the intervention group facilities. Experience from the pilot study demonstrated that this usual method of documenting personal care using a flowsheet can be an effective and efficient means to assess the sit-to-stand activity adherence of both the health care aides and the residents. We will use this usual documentation as a research data collection tool to minimize the response burden for health care aides. The research assistant will conduct spot checks to compare health care aide performance of the sit-to-stand activity with what they record on the flowsheets. The flowsheets will be collected monthly for the duration of the study.

The frequency and intensity of practicing the sit-to-stand activity will be abstracted from the flowsheets every month in the intervention facilities. The number of occasions per month that health care aides document that the functional activity is completed will be the measure of the frequency. The mean number of sit-to-stands completed on each occasion will be the measure of performance intensity. If after one month it is apparent that the residents in the intervention group are not performing the sit-to-stand activity at least once a day then we will conduct brief interactive education sessions with the health care aides to review the procedure for the sit-to-stand activity and the documentation process and to identify strategies to overcome any reported barriers.

## Measures

We are interested in functional activity which, according to the International Classification of Functioning, Disability and Health [55] framework, involves the execution of tasks or actions at the level of the whole human being in the daily life of the individual; therefore, we will measure functional outcomes rather than balance and strength which more narrowly focus on body functions and structure.

### Outcomes

#### Mobility

The sit-to-stand action is a functional activity that has been incorporated into a number of mobility measures including the time to rise without using arms, time for 5 sit-to-stands, time for 10 sit-to-stands, and the number of sit-to-stands completed in 30 seconds. We have chosen to measure mobility using the number of sit-to-stands in 30 seconds because in the frail nursing home population many residents must use their arms to stand [10,38,47,48] and may be unable to complete more than one sit-to-stand. This mobility assessment will be completed by a research assistant using a stopwatch and standard armchair with a standard height of 42 cm. Residents will be instructed to stand up and sit down as many times as possible until they are asked to stop after 30 seconds. In a group of community-dwelling older adults aged 66 to 97 years [47], researchers found evidence for test-retest reliability, criterion validity (comparing the chair stand performance to a measure of lower body strength), and discriminant validity (comparing performance in different age and physical activity groups) with the 30-second sit-to-stand test.

#### Function

The Functional Independence Measure (FIM) is an 18-item instrument which assesses the amount of assistance required to complete basic activities of daily living using a 7-point scale graded from 1 (dependent) to 7 (independent) [56-58]. The FIM includes motor (self-care, sphincter control, transfers, locomotion) and cognitive (communication and social-cognition) subscales. The 18 individual items are totalled to produce a score ranging from 18 to 126. Originally designed with rehabilitation populations in mind [57], the reliability and validity of the FIM has been assessed with older adults in acute care [59] and the community [58,60] with demonstrated reliability in each of these settings [61]. More recently the FIM has been used in a LTC setting [21,62,63] and has been compared with the Resident Assessment Instrument [64-68]. Eight responsiveness studies all found the physical but not the cognitive subscales to be responsive to change [59,65]. We will interview health care aides using a structured questionnaire to measure residents' functional abilities.

#### Health-Related Quality of Life

The Health Utilities Index Mark 2 and 3 (HUI2/3) is a generic health-related quality of life questionnaire based on two generic multi-attribute preference-based systems: the HUI2 and the HUI3 [69]. Interview administered versions of the HUI2/3 can typically be completed in 3 minutes [70] although other researchers report that it takes 15 to 20 minutes to administer the HUI2 to frail older adults [71]. The HUI2 assesses capacity on six dimensions (or attributes) of health status: sensation (vision, hearing, and speech), mobility, emotion, cognition, self-care, and pain. The HUI3 assesses capacity on eight attributes: vision, hearing, speech, ambulation, dexterity, emotion, cognition, and pain with each attribute consisting of five or six levels ranging from normal to severely impaired function. The HUI3 may be less prone to floor effects than the HUI2, and the HUI3 has a greater capacity to measure a wider range of utility scores than does the HUI2. Together the HUI2 and HUI3 provide complementary and clinically relevant information for LTC residents with dementia. Both have been used in samples of people with dementia [72-75]. When the HUI2 was administered to frail older adults and caregiver proxy respondents of people with dementia there was evidence of discriminant validity [76], construct validity [74] and responsiveness to decline but not responsiveness to improvement [72,74]. A change of 0.03 or more in overall HUI scores is considered clinically important as is a change in level within any of the HUI attributes [71]. The HUI2/3 was chosen over other health-related quality of life measures because its widespread use facilitates the interpretation of results and allows comparisons.

#### Dementia Specific Quality of Life

Because utility based instruments are generic measures, a measure of attributes specific to residents with dementia is also needed [76]. We have selected the caregiver version of the Quality of Life - Alzheimer's Disease (QOL-AD) instrument because it has excellent psychometric properties, is brief (generally administered in 10 minutes), and is based on a sound conceptual framework. The QOL-AD was developed to capture the domains considered important to health-related quality of life in Lawton's broad conceptual framework: the interpersonal; environmental; functional; physical; and, psychological domains [77,78]. The 13 items including physical health, energy, mood, living situation, memory, family, marriage, friends, self as a whole, ability to do chores around the house, fun, money and life as a whole scored on a 4-point Likert scale ranging from 1 (poor) to 4 (excellent). Total scores range from 13 to 52 with 6 points on the QOL-AD scale equivalent to 1 standard deviation. The instrument has demonstrated responsiveness to change [79,80] and good evidence of reliability (coefficient α = 0.87; ICC = 0.92) and validity [80-83]. Caregiver ratings on the QOL-AD were not correlated with various levels of the person with dementia's cognitive functioning. Criterion related validity was demonstrated with Pearson correlation coefficients for measures of behavioural competence, psychological status, physical function and interpersonal environment [82,84]. Some individual item scores for the person with dementia do not correlate with those of the proxy caregiver [82,84].

### Covariates

Depression, cognitive impairment, and comorbidities have been identified as important predictors of loss of mobility [85-87]. Measures for each of these covariates can be derived from the Resident Assessment Instrument 2.0 (RAI 2.0) [88-90] which is completed quarterly in all Alberta LTC facilities. We have opted to use these data for some of the covariate measures to minimize response burden. For each resident participant we will acquire the RAI 2.0 data directly from the LTC facility for the three quarters that best approximate the times in which resident's baseline, three month and six month outcome data are collected. We will use the following scales derived from the RAI 2.0: the *MDS Depression Rating Scale (DRS) *[91,92]; the *Cognitive Performance Scale (CPS) *[93-95]; and, the *Changes in Health, End-stage disease Symptoms & Signs Scale (CHESS) *[96].

The degree to which the environment supports the person with dementia has been identified as an important predictor of loss of mobility [4]; therefore we will use the *Professional Environmental Assessment Protocol *(PEAP) and the *Alberta Context Tool *(ACT) as covariates. The PEAP provides a global assessment of the quality of dementia care environments on nine dimensions deemed to be therapeutic for people with dementia: awareness and orientation, safety and security, provision of privacy, regulation of stimulation, quality of stimulation, support of functional abilities, opportunities for personal control, facilitation of social contact, and continuity of the self with the past through personal and familiar objects. It involves a subjective evaluation of the physical and social environment on a 13-point scale for each dimension, with total scores ranging from 13 to 117 [97]. All PEAP dimensions have demonstrated good inter-rater reliability: percentage agreement from 58.3 to 91.7%, Spearman's from 0.69 to 0.88 and kappas from 0.69 to 0.85 [98]. The PEAP total scores correlate with the more established Therapeutic Environment Screening Scale (r = 0.89) [99] providing evidence for criterion-related validity [100]. Correlations among the PEAP dimensions ranged from 0.45 to 0.85 and a principal components analysis generated a single factor structure for the nine PEAP dimensions accounting for 67% of the total variance [101]. The PEAP summary scores discriminated between special care units and integrated facilities in rural Canadian LTC facilities [101].

The Alberta Context Tool (ACT) which measures organizational context in health care settings was developed to predict research utilization. It consists of eight dimensions that are potentially modifiable: culture, leadership, evaluation, social capital, informal interactions, formal interactions, structural and electronic resources, and organizational slack [102]. The ACT comprises 56 multiple choice items scored on five-point scales with the exception of the structural and electronic resources items scored on a six-point scale. Psychometric properties of the measure were assessed with a sample of health professionals in six Canadian pediatric hospitals [103]. A principal components analysis generated a 13 factor structure accounting for 59% of the total variance. Correlations among the 13 ACT factors ranged from 0.54 to 0.91providing evidence for internal consistency. Instrumental research utilization [103] was correlated with 12 of the 13 ACT factors at the 5% level of significance providing evidence of construct validity.

Facility level characteristics such as size of facility, size of unit and ownership model (public, private for-profit, voluntary not-for-profit) are also potential predictors of resident outcome.

### Data Analysis

All data will be entered into an SPSS 19 database (IBM, 2010, ^©^IBM Corporation). Ten percent of the data will be double entered to assess the accuracy of data entry. We will compare the baseline characteristics of the LTC facilities, health care aides, and residents in the intervention group with the control group by computing descriptive statistics. Data are structured for each outcome measure (the 30-second sit-to-stand test; the *Functional Independence Measure*; the *Health Utilities Index Mark 2 and 3; *and, the *Quality of Life - Alzheimer's Disease*) to allow a mixed between-within subjects analysis of covariance. Specifically, the between subjects factor consists of the two groups (Control versus Intervention Group) and the within subject factor consists of the repeated measures. The covariates will include baseline characteristics the residents, including age, sex, level of cognitive impairment (Cognitive Performance Scale), extent of depression (MDS Depression Rating Scale), and extent of health instability (CHESS), and characteristics of the LTC facilities including facility size, unit size, ownership model, and quality of the environment (PEAP and ACT). A Multivariate Analysis of Covariance (MANCOVA) will be used to control the experiment-wise error rate for comparisons. Data will be analyzed according to the intention-to-treat principle, i.e. residents who relocate to another setting or who die before completing the six months of follow-up will be included in the data analysis. The extent to which the residents in the intervention facilities actually received the mobility interventions will be assessed using the documentation flowsheet measures to establish a graphical analysis of the covariate-corrected time course of performance on the mobility intervention. These analyses will be conducted using SPSS 19 in consultation with a statistician from the Centre of Health Promotion Studies at the University of Alberta.

### Ethical Considerations

We have received ethical approval for the study from the Health Research Ethics Board of the University of Alberta. We also have received a letter of support from the senior administrator of each of the six participating LTC facilities.

#### Adverse Events

Monitoring of adverse events in the intervention facilities will be compared with adverse events in the control facilities. We expect to see a reduced incidence of resident falls and related injuries in the intervention facilities. Likewise we expect to see a reduction in the injury of health care aides working in the intervention facilities. Resident safety is unlikely to be compromised given our pilot study experience and the experience of others in carrying out exercise interventions in a similar population [20,28,37,42,104,105]. Although the mobility intervention is expected to reduce residents' risk of falls by maintaining or improving their mobility, maintaining mobility extends the time that residents are at risk of a fall. We will monitor resident participants' falls monthly and compare these with their baseline pattern of falls three months prior to entering the study. In the event of a resident participant falling in one of the intervention facilities the principal investigator will decide in collaboration with the LTC facility manager whether or not that resident should remain in the study.

### Knowledge Translation

We have engaged knowledge users from the outset of the project to enhance the likelihood of the uptake and sustainability of the innovation. This is consistent with the two communities theory which emphasizes the importance of bridging the gap between researchers and knowledge users by engaging knowledge users early in the research process [106] and with the literature suggesting that managers attitudes toward an innovation have a strong impact on the adoption of the innovation [107]. The knowledge users and applicants will meet frequently at the beginning of the project and then bimonthly to monitor the progress of the research and provide advice on issues as they inevitably arise. At the end of the study we will host a knowledge translation symposium in which the investigators, the knowledge users, collaborators, and other interested stakeholders will be invited to assess the relevance of the study findings in light of related evidence, the potential impact, and methods to extend the application of the findings. The symposium will provide a forum for a full exchange of ideas in light of the evidence generated from this study and the existing literature. It is in the context of the synthesized evidence that decisions will be made about if and how to integrate the new knowledge into practice and/or policy.

## Discussion

The findings of this study will provide clinically relevant information on the effect of the sit-to-stand functional activity on mobility, function and health-related quality of life among individuals with dementia. Not only will it provide resident-specific information but also how and if health care aides are willing and able to integrate the activity into their daily work with residents; and what might be done to translate these findings into practice to improve care for LTC residents with dementia. The sit-to-stand mobility intervention introduces change at the level of the LTC facility, the health care team, the individual health care aide, and the resident [108].

This project represents an interdisciplinary partnership between knowledge users and new and established investigators, with a focus on maintaining the mobility, function and health-related quality of life of LTC residents. There are several compelling reasons for this study: the widespread prevalence of limited mobility in this population, the rapid decline in mobility after admission to a LTC facility, the critical importance of mobility to quality of life, the increased time (and therefore cost) required to care for residents whose mobility is compromised, and the increased risk of injury for health workers who are providing care for residents unable to stand. The importance of these issues is magnified when considering the increasing number of people living in long-term care facilities and an aging population. Demonstrating a feasible and practical mobility intervention with the potential to improve or maintain functional mobility in frail and vulnerable older Canadians in LTC facilities, and to reduce the risk of injury to direct care providers is a safety, economic and, importantly, a quality of life issue.

## List of Abbreviations

The list of abbreviations contained in this article are as follows: Functional Independence Measure (FIM); Health Utilities Index Mark 2 and 3 (HUI2/3); Health Utilities Index 2 (HUI2); Health Utilities Index 3 (HUI3); long-term care (LTC); Quality of Life - Alzheimer's Disease (QOL-AD); intraclass correlation (ICC); Resident Assessment Instrument 2.0 (RAI 2.0); Depression Rating Scale (DRS); Cognitive Performance Scale (CPS); Changes in Health; End-stage disease Symptoms and Signs Scale (CHESS); Professional Environmental Assessment Protocol (PEAP); Alberta Context Tool (ACT) and Multivariate Analysis of Variance (MANCOVA).

## Competing interests

The authors declare that they have no competing interests.

## Authors' contributions

SES conceived of the study, led in the development of this protocol and prepared the initial draft the manuscript. CAE, CAJ and ASW participated in the development of the study design and revisions of the protocol. CAJ advised on the selection of outcome measures. All authors read and approved the final manuscript.

## Pre-publication history

The pre-publication history for this paper can be accessed here:

http://www.biomedcentral.com/1471-2318/11/84/prepub
